# Allogeneic Hematopoietic Cell Transplantation for Congenital Athymia: A Nationwide Retrospective Study in Japan

**DOI:** 10.1007/s10875-026-02008-y

**Published:** 2026-03-28

**Authors:** Tsubasa Nishinosono, Hideki Muramatsu, Manabu Wakamatsu, Motoshi Sonoda, Katsuhide Eguchi, Koji Kawaguchi, Takeshi Yamamoto, Takahiro Kudo, Michiko Kajiwara, Masataka Ishimura, Yoshiyuki Takahashi

**Affiliations:** 1 Department of Pediatrics, Nagoya University Graduate School of Medicine, 65 Tsurumai-cho, Showa-ku, Nagoya, Aichi, 466-8560 Japan; 2 Department of Pediatric Critical Care, Shizuoka Children’s Hospital, Shizuoka, Japan; 3 Department of Pediatrics, Graduate School of Medical Sciences, Kyushu University, Fukuoka, Japan; 4 Department of Hematology and Oncology, Shizuoka Children’s Hospital, Shizuoka, Japan; 5 Department of Pediatrics, Graduate School of Medicine, Chiba University, Chiba, Japan; 6 Department of Pediatrics, Juntendo University Faculty of Medicine, Tokyo, Japan; 7 Center for Transfusion Medicine and Cell Therapy, Institute of Science Tokyo Hospital, Tokyo, Japan

**Keywords:** Congenital athymia, Complete DiGeorge syndrome, 22q11.2 deletion syndrome, CHARGE syndrome, Hematopoietic cell transplantation

## Abstract

**Purpose:**

Congenital athymia is a life-threatening condition characterized by thymic absence and profound T-cell immunodeficiency. Thymus implantation is the definitive treatment, but its availability is limited. This study aimed to evaluate the outcomes of hematopoietic cell transplantation (HCT) as an alternative therapy.

**Methods:**

A nationwide, multicenter, retrospective study analyzed nine patients who underwent allogeneic HCT between 2000 and 2024 in Japan. Overall survival (OS) was estimated using the Kaplan–Meier method, and the cumulative incidence of immunodeficiency-related mortality was assessed using Gray’s test.

**Results:**

Among the nine patients, eight (89%) received umbilical cord blood, and one (11%) received related peripheral blood. Seven patients (78%) underwent transplantation without conditioning. Engraftment with T-cell recovery was achieved in six patients (67%), with a median CD4^+^ T-cell count of 0.352 × 10^9^/L (range, 0.216–1.578 × 10^9^/L) at the last follow-up. Acute graft-versus-host disease (GVHD) occurred in five patients (56%), all with Grade I–II skin involvement. No chronic GVHD was observed. The one-year OS rate was 66.7% (95% confidence interval: 28.2%–87.8%). Overall, six patients (67%) died: three early deaths within the first month from infections, and three late deaths beyond 1 year from congenital comorbidities. HCT before six months of age was associated with significantly lower immunodeficiency-related mortality (*p* = 0.02).

**Conclusion:**

HCT can allow immune reconstitution in congenital athymia, although long-term survival is affected by comorbidities. Early diagnosis and timely intervention are crucial in managing this condition. HCT can be a restricted alternative therapy for patients ineligible for thymus implantation.

**Supplementary Information:**

The online version contains supplementary material available at 10.1007/s10875-026-02008-y.

## Introduction

Congenital athymia is characterized by the absence of a thymus at birth, resulting in profound T-cell immunodeficiency and an increased susceptibility to life-threatening infections [1, 2]. It is primarily linked to genetic disorders such as complete DiGeorge syndrome (cDGS), 22q11.2 deletion syndrome, and CHARGE syndrome. CHARGE syndrome, caused by *CHD7* variants, is a complex condition with various abnormalities including coloboma, congenital heart defects, choanal atresia, growth retardation, and hearing impairment. It is often associated with thymic hypoplasia or aplasia, both of which contribute to varying degrees of immunodeficiency [3–5]. DGS is characterized by thymic hypoplasia, congenital heart defects, hypoparathyroidism, and multiple craniofacial anomalies [6–8]. Notably, cDGS presents as severe combined immunodeficiency (SCID) with complete thymic absence and profound T-cell lymphopenia, necessitating prompt diagnosis and intervention to prevent fatal infections [9]. In cDGS, chromosome 22 deletions account for 52% of cDGS cases, while *CHD7* variants are responsible for 26% [10].

Advances in newborn screening (NBS) using T-cell receptor excision circle (TREC) assays have significantly enhanced the early detection of congenital T-cell immunodeficiencies, such as SCID and cDGS [11–14]. TRECs are reliable biomarkers of thymic function and naïve T-cell output, enabling the identification of affected individuals before the onset of infections. Early diagnosis through NBS has proven life-saving by facilitating timely interventions, such as hematopoietic cell transplantation (HCT) for SCID [15–17] and thymus implantation for cDGS [11].

Thymus implantation is the definitive treatment for congenital athymia, but its availability is limited due to logistical and clinical challenges [2, 10, 18]. This procedure is offered at only two centers, one in the United States and the other in the United Kingdom, and is not performed in Japan. In settings where thymus implantation is unavailable or contraindicated, HCT has been employed as an alternative therapy to reconstitute T-cell immunity [19–21]. The long-term efficacy of HCT in terms of immune reconstitution and survival is still uncertain. This study aimed to assess the outcomes and prognosis of patients with congenital athymia who underwent allogeneic HCT in Japan. A nationwide, multicenter, retrospective study provides valuable insight into the safety and efficacy of HCT, particularly as an alternative in resource-limited settings without access to thymus implantation.

## Methods

### Data Collection

This retrospective study analyzed patients with congenital athymia who underwent allogeneic HCT between April 2000 and March 2024 in Japan. Clinical data from nine patients treated at six centers were collected through questionnaires distributed to 68 centers affiliated with the Japanese Society for Immunodeficiency and Autoinflammatory Diseases. Partial clinical information on six of the nine patients had been previously published as individual case reports [21–25]. In the present nationwide study, all transplant-related variables, immunologic data, and follow-up information were newly collected from each participating institution via a standardized survey and analyzed as a single dataset. Congenital athymia was defined as the complete absence of the thymus, confirmed by chest radiography, magnetic resonance imaging, or direct visualization during surgery. The diagnosis of cDGS was established when athymia coexisted with congenital heart disease and/or hypoparathyroidism. The 22q11.2 deletion syndrome was genetically diagnosed by detecting a 22q11.2 deletion. CHARGE syndrome was diagnosed based on clinical features and confirmed by identifying pathogenic *CHD7* variants. T-cell lymphopenia due to athymia was defined as a total CD3^+^ T-cell count below 0.05 × 10^9^/L or a CD45RA^+^ naïve T-cell count below 0.05 × 10^9^/L.

### Statistical Analysis

This retrospective study aimed to evaluate survival outcomes to determine the efficacy of allogeneic HCT in patients with congenital athymia. Overall survival (OS) was defined as the duration of survival from birth without mortality. The cumulative incidence of immunodeficiency-related mortality was estimated, with immunodeficiency-related death (e.g., death due to infection) considered as the primary event of interest. Deaths from comorbidities unrelated to immunodeficiency (e.g., congenital heart disease or airway disorders) were considered as competing risks. Patients who were still alive at the last follow-up were censored at that time. Survival probabilities were estimated using the Kaplan-Meier method, with survival rates calculated from birth until either death or the last observation. Differences between cumulative incidence curves were assessed using Gray’s test. Continuous variables were compared using the Mann-Whitney *U* test, while categorical variables were compared using Fisher’s exact test. All statistical analyses were conducted using EZR (Saitama Medical Center, Jichi Medical University, Saitama, Japan), a graphical user interface for R software version 4.3.1 (The R Foundation for Statistical Computing, Vienna, Austria) [26].

### Ethics Statement

Written informed consent or opt-out consent was obtained from all patients or their parents prior to their participation in the study. The study protocol was approved by the Ethics Committee of the Nagoya University Graduate School of Medicine.

## Results

### Patient Characteristics

The baseline characteristics of the nine patients are summarized in Table 1. The median age at diagnosis was 54 days (range, 30–335 days), with one patient identified *via* NBS. None of the cases had a family history of congenital athymia. Genetic defects were identified as *CHD7* variants in six patients, 22q11.2 deletion in two, and unknown genetic etiology in one presenting clinically with cDGS.

**Table 1:** Patient characteristics

UPN	Gender	Age at the time of diagnosis, days	Identified by NBS	Phenotype	22q11.2 deletion	*CHD7 *variant	Congenital heart disease	HypoPTH	Airway disorders	Pre-transplant data				Infection before HCT	Ref
										CD3^+^ cells,× 10^9^/L	CD45RA^+^ cell, × 10^9^/L	Mitogen response (PHA)	TREC		
1	M	101	–	cDGS	NA	–	–	Y	–	0	NA	NA	NA	Systemic CMV infection (interstitial pneumonia, gastroenteritis, hepatitis, and retinopathy)	[22]
2	M	30	–	CHARGE	*–*	c.1036 A > T, p.Arg346Ter	TA, IAA, ARSA	Y	Y	0.008	NA	Low	Undetected	–	[21, 23]
3	F	55	–	CHARGE	*–*	c.2507-2510delTTCA, p.His837ValfsTer5	PDA	Y	Y	0.040	NA	Low	NA	–	[21]
4	F	NA	–	22qDS	Y	–	ARSA, RAA	Y	Y	0.043	NA	NA	NA	Severe pneumonia and ARDS with PIV3	*–*
5	M	335	–	CHARGE	*–*	c.3106 C > T, p.Arg1036Ter	ASD	Y	Y	0.080	0	NA	Undetected	*Enterobacter aerogenes* urinary tract infection	*–*
6	F	73	–	22qDS	Y	–	VSD	Y	Y	0.026	0	Low	Undetected	Pneumonia	[24]
7	F	52	–	CHARGE	*–*	c.469 C > T, p.Arg157Ter	HLHS, CoA, VSD, ASD	Y	Y	0	0	NA	Undetected	*Pseudomonas spp.* respiratory tract infection	[21, 25]
8	F	36	Y	CHARGE	*–*	c.2572 C > T, p.Arg858Ter	PDA	Y	Y	0	0	NA	Undetected	–	[21]
9	M	50	–	CHARGE	*–*	deletion 8q11.2-q12.3	PDA	Y	Y	0.036	NA	Low	NA	Otitis media and pneumonia	–

*22qDS* 22q11.2 deletion syndrome, *ARDS* acute respiratory distress syndrome, *ARSA* aberrant right subclavian artery, *ASD* atrial septal defect, *cDGS* complete DiGeorge syndrome, *CMV* cytomegalovirus, *CoA* coarctation of the aorta, *HCT* hematopoietic cell transplantation, *HLHS* hypoplastic left heart syndrome, *HypoPTH* hypoparathyroidism, *IAA* interrupted aortic arch, *NA* not available, *NBS* newborn screening, *PDA* patent ductus arteriosus, *PHA* phytohemagglutinin, *PIV3* parainfluenza virus type 3, *RAA* right aortic arch, *TA* Truncus arteriosus, *TREC* T-cell receptor excision circle, *UPN* unique patient number, *VSD* ventricular septal defect

Congenital heart disease was present in eight patients (89%), including severe cases requiring early surgical intervention, such as hypoplastic left heart syndrome and truncus arteriosus with interrupted aortic arch. All patients (100%) had hypoparathyroidism, necessitating calcium and/or vitamin D supplementation. Airway disorders were observed in eight patients (89%), with six patients with CHARGE syndrome requiring tracheostomy. Neuropsychiatric disorders, such as developmental disabilities and epilepsy, were noted in eight patients (89%). Pre-transplant infections, including systemic cytomegalovirus (CMV) infection and severe pneumonia due to parainfluenza virus type 3 (PIV3), were experienced by six patients (67%). Detailed congenital comorbidities for each patient are summarized in Table S1.

Pre-transplant immunological evaluations are summarized in Table S2. Median T-cell counts were 0.026 × 10^9^/L (range: 0–0.080 × 10^9^/L) for CD3^+^, 0.003 × 10^9^/L (range: 0–0.071 × 10^9^/L) for CD4^+^, and undetectable (range: 0–0 × 10^9^/L) for CD4^+^CD45RA^+^. All patients demonstrated a low mitogen response to phytohemagglutinin and undetectable TREC levels.

### Transplantation

The transplantation details and outcomes are summarized in Table 2. Allogeneic HCT was conducted at a median age of 165 days (range, 70–358 days). Eight patients (89%) received unrelated cord blood, while one (11%) received related peripheral blood from the mother. Seven patients (78%) underwent transplantation without conditioning, while two (22%) received conditioning regimens: fludarabine plus busulfan (UPN 5) and fludarabine plus melphalan (UPN 6). All patients received graft-versus-host disease (GVHD) prophylaxis: tacrolimus plus methotrexate (*n* = 7), tacrolimus alone (*n* = 1), and cyclosporine (*n* = 1).

**Table:** 

UPN	HCT						Complications				Outcomes	
	Age at HCT, days	Graft source	HLA matching	Conditioning	GVHD prophylaxis	Engraftment	Acute GVHD, grade	Chronic GVHD	Others	Infection after HCT	Survival status, post-HCT	Cause of death
1	226	uCBT	NA		CyA	–	–	–	–	CMV reactivation	Dead, Day 30	CMV reactivation
2	144	uCBT	8/8	–	Tac, sMTX	Y	I	–	–	–	Dead, Month 16	Respiratory failure and arrhythmia
3	107	uCBT	6/8	–	Tac, sMTX	Y	I	–	–	–	Dead, Month 20	Sudden cardiac arrest
4	207	rPBSCT	5/8	–	Tac	–	–	–	–	–	Dead, Day 3	Secondary pulmonary alveolar proteinosis due to PIV3
5	358	uCBT	6/8	FLU, BU	Tac, sMTX	–	–	–	–	Y (Unknown)	Dead, Day 14	Septic renal and cardiac failure
6	165	uCBT	4/8	FLU, MEL	Tac, sMTX	Y	–	–	ITP	Aspiration pneumonia	Alive, Month 131	–
7	71	uCBT	7/8	–	Tac, sMTX	Y	II	–	Stroke due to protein C deficiency	Catheter infection	Dead, Month 17	Progressive heart failure
8	70	uCBT	7/8	–	Tac, sMTX	Y	II	–	–	Catheter infection	Alive, Month 37	–
9	189	uCBT	7/8	–	Tac, sMTX	Y	II	–	–	–	Alive, Month 28	–

*BU* busulfan, *CMV* cytomegalovirus, *CyA* cyclosporine A, *FLU* fludarabine, *GVHD* graft-versus-host disease, *HCT* hematopoietic cell transplantation, *ITP* immune thrombocytopenic purpura, *MEL* melphalan, *MTX* methotrexate, *NA* not available, *PIV3* parainfluenza virus type 3, *rPBSCT* related peripheral blood stem cell transplantation, *Tac* tacrolimus, *uCBT* unrelated cord blood transplantation, *UPN* unique patient number

Six patients (67%) achieved engraftment, while three (33%) died of infections within the first month post-transplantation: CMV reactivation, sepsis of unknown origin, and secondary alveolar proteinosis due to pre-transplant PIV3 infection. Engrafted patients exhibited mixed donor T-cell chimerism (range, 59%–95%), assessed using short tandem repeat or fluorescence *in situ* hybridization analysis. Acute GVHD occurred in five patients (56%), all with Grade I or II skin involvement, with no cases of chronic GVHD observed.

### Clinical Course and Survival

**Fig. 1:**
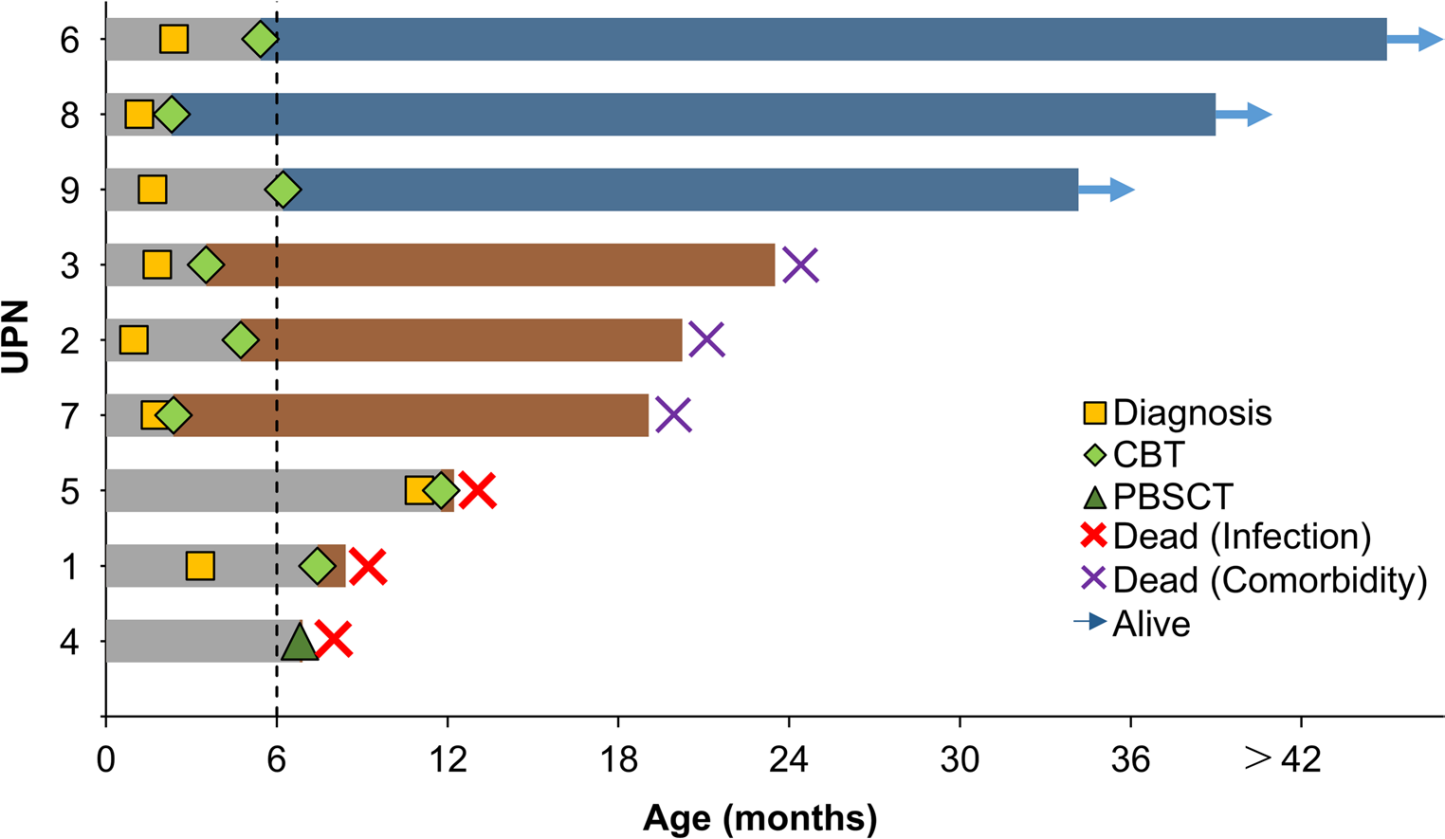
Clinical course of patients with congenital athymia. The swimmer plot illustrates the clinical course of nine patients with congenital athymia. Immunodeficiency-related mortality occurred due to infections within the first month post-hematopoietic cell transplantation (HCT), while other patients died of underlying comorbidities during the late phase, defined as beyond one year post-HCT. Three patients who died of infections received HCT after six months of age, and they were significantly older at the time of HCT compared to those who survived beyond one year (*p* = 0.02). CBT, cord blood transplantation; UPN, unique patient number; PBSCT, peripheral blood stem cell transplantation

The clinical courses of the nine patients are illustrated in Fig. 1. During the observation period, six patients (67%) died. Three died early (within the first month after HCT) from immunodeficiency-related infections, whereas the remaining three died later (over 1 year) due to non-immunodeficiency-related congenital comorbidities. The late deaths were due to progressive heart failure in a patient with hypoplastic left heart syndrome (UPN 7), arrhythmia following recurrent episodes of respiratory distress in a patient with truncus arteriosus/interrupted aortic arch on home mechanical ventilation (UPN 2), and sudden cardiac arrest (UPN 3).

**Fig. 2:**
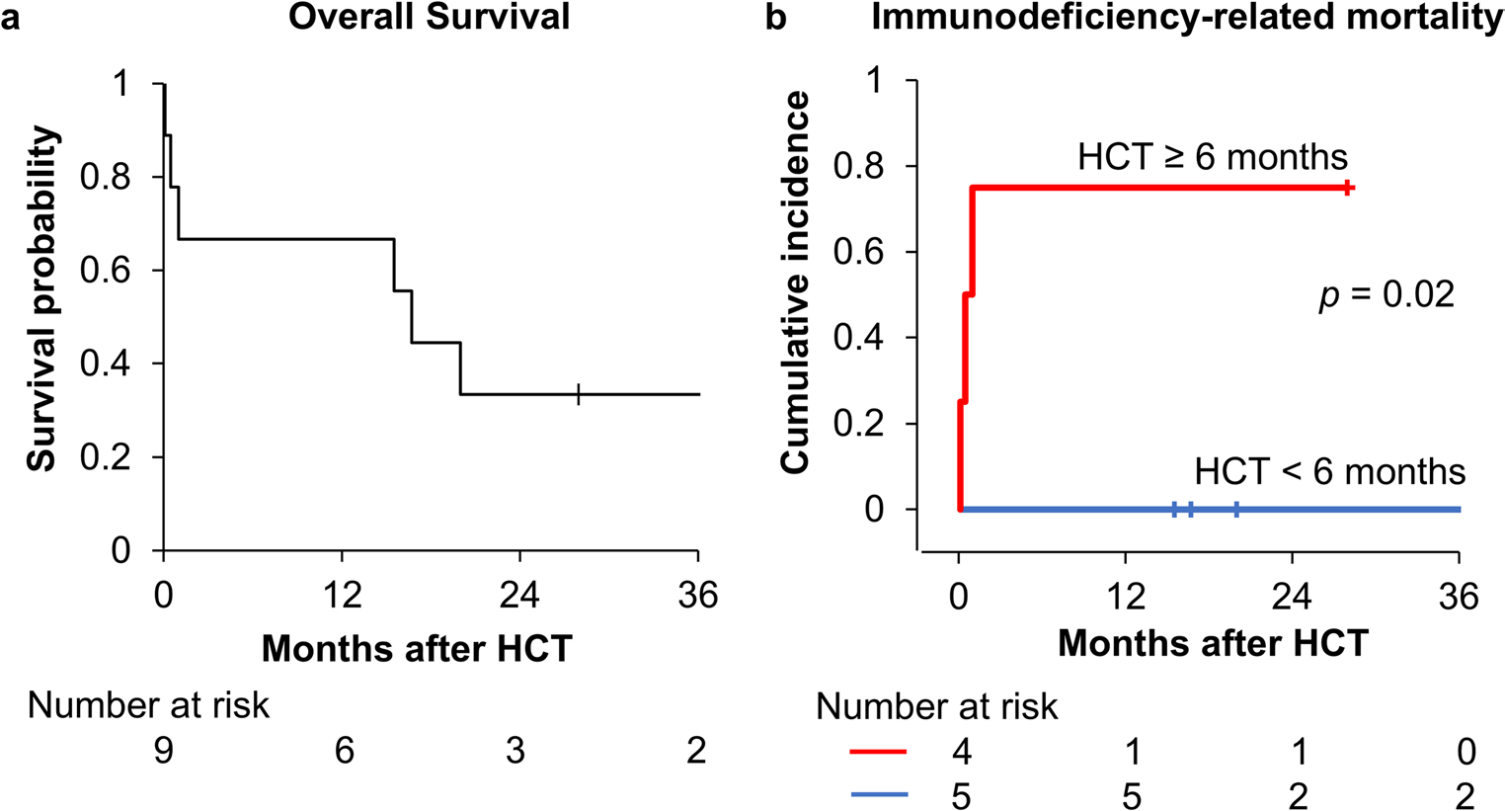
Overall survival and immunodeficiency-related mortality in patients with congenital athymia after hematopoietic cell transplantation (HCT). (**a**) The Kaplan–Meier overall survival curve for nine patients with congenital athymia demonstrates a one-year overall survival rate of 66.7%. (**b**) Cumulative incidence curves for immunodeficiency-related mortality reveal that HCT before six months of age was associated with a significantly lower incidence of immunodeficiency-related mortality compared to later HCT (0% vs. 75%; *p* = 0.02). Deaths from other causes, such as comorbidities, were considered as competing risks

Among the six patients who achieved T-cell reconstitution, three experienced late deaths, whereas the remaining three survived with immune recovery. The one-year OS rate was 66.7% (95% confidence interval: 28.2%–87.8%), with a median follow-up of 1.39 years (range, 0.01–10.9 years). Patients who died of infection, classified as immunodeficiency-related deaths, were older at the time of transplant than survivors (*p* = 0.02). All three early immunodeficiency-related deaths occurred exclusively in patients with pre-transplant infections. HCT performed before six months of age was associated with a significantly lower cumulative incidence of immunodeficiency-related mortality compared to HCT performed later (0% vs. 75%; *p* = 0.02, Fig. 2).

### Immune Reconstitution

**Fig. 3:**
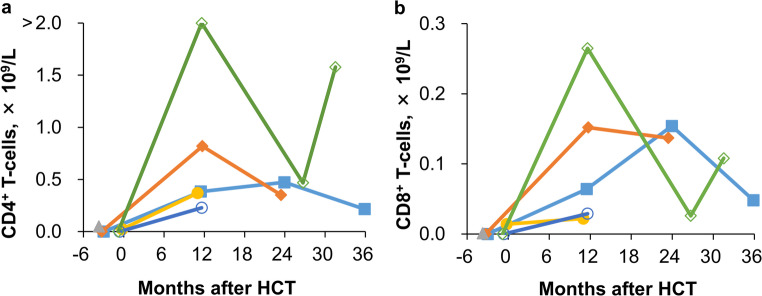
Immune reconstitution in patients with congenital athymia after hematopoietic cell transplantation (HCT). Post-transplant immunological assessment in long-term survivors demonstrated a progressive increase in (a) CD4+ and (b) CD8+ T-cell counts over time post-HCT. Data from five survivors are included in the analysis, as data for one case were missing

Post-transplant immune reconstitution was assessed in the six long-term survivors. Changes in CD4^+^ and CD8^+^ T-cell counts over time are illustrated in Fig. 3, with data available for five cases as one case had missing data. The median CD4^+^ T-cell count at the last follow-up was 0.352 × 10^9^/L (range, 0.216–1.578 × 10^9^/L). Post-transplant immunoglobulin replacement therapy (IRT) duration and antibody responses are detailed in Table S3. Among the survivors, two (33%) did not receive post-transplant IRT, while four (67%) received regular IRT, with two discontinuing it within one year. Vaccinations elicited immune responses in three patients (50%): two developed antibodies against hepatitis B surface antigen, and one against diphtheria, poliovirus, and pertussis.

## Discussion

This nationwide retrospective study in Japan evaluated the outcomes of HCT for congenital athymia. Although HCT promoted immune reconstitution in a subset of patients, overall survival remained limited, with six of the nine patients ultimately dying during the study period. Early deaths resulted from immunodeficiency-related infections, whereas later deaths occurred due to severe congenital comorbidities despite immune reconstitution.

The increased mortality rates observed in patients transplanted after six months of age underscore the critical importance of early diagnosis and timely intervention. All early immunodeficiency-related deaths in our cohort occurred among patients with pre-transplant infections. In SCID, early HCT before four months of age has been shown to enhance survival by reducing infection risks through NBS [16, 27, 28]. Similarly, early thymus implantation for congenital athymia before four months of age has been associated with lower infection rates [11]. These findings emphasize the necessity of early detection and prompt HCT not only for SCID but also for congenital athymia. Within this context, one patient in our cohort (UPN 8) was identified through TREC-based NBS and underwent cord blood transplantation at 70 days of age, prior to the development of infections, and achieved long-term survival with immune reconstitution. This case illustrates how early diagnosis and intervention may translate into favorable immune outcomes in patients with congenital athymia.

A distinct feature of this nationwide cohort is the higher proportion of patients who underwent cord blood transplantation, with eight of the nine patients receiving cord blood. This finding comes in contrast with previous case series on HCT for congenital athymia, which predominantly used bone marrow or peripheral blood stem cells. In the largest multicenter cohort to date, only one patient received cord blood transplantation, with an overall survival rate of 41% [20]. In the mentioned multicenter cohort study, more than half of patients developed clinically significant acute GVHD (Grade II–IV), and a subset of these patients subsequently developed chronic GVHD, which contributed to morbidity and mortality, potentially due to the absence of thymus-derived regulatory T cells [20]. In contrast, the current study observed only mild GVHD (Grade II or lower), with none of our patients developing chronic GVHD. Although causal inference is limited, the predominance of cord blood transplantation may have contributed to this favorable GVHD profile.

Thymus implantation serves as the definitive treatment for congenital athymia, with reported survival rates ranging from 75% to 80% [2, 11, 18, 29]. However, its application is restricted by the scarcity of specialized centers, strict eligibility criteria, and the prerequisite of stable cardiopulmonary function. Post-implantation mortality is primarily attributed to infections within the first year [2, 18]. Successful thymus implantation necessitates effective infection control during the peri-implantation period (6–12 months), which poses challenges for patients with severe infections or cardiopulmonary instability [30, 31]. Furthermore, thymus implantation is contraindicated in children requiring prolonged surgery, mechanical ventilation, or corticosteroids, commonly used in cardiac or airway procedures [30, 31]. In clinical contexts where thymus implantation is not feasible due to severe clinical conditions, HCT has been explored as a therapeutic option in selected patients [32, 33]. In our cohort, two patients with single-ventricle physiology (UPN 2 and UPN 7) survived beyond 1 year after non-conditioning cord blood transplantation without immunodeficiency-related mortality.

This study has several limitations. First, the small sample size and retrospective design restricted the assessment of the impact of donor sources, conditioning regimens, genotype, and comorbidities on outcomes. Although conditioning could theoretically support engraftment by reducing residual host immune function, its benefit in congenital athymia remains uncertain. Most of the patients in our cohort had severe cardiopulmonary or airway comorbidities or active infections, likely decreasing their tolerance to cytotoxic conditioning. Moreover, only two patients received conditioning, precluding meaningful comparison. Second, only one patient in this study was identified through NBS. As more cases are identified through NBS, research is necessary to determine the most effective timing and approach for HCT in congenital athymia.

In conclusion, HCT allowed immune reconstitution in the surviving patients with congenital athymia, although severe underlying congenital comorbidities constrained long-term survival. For patients ineligible for thymus implantation due to cardiopulmonary instability or limited access, HCT may serve as a restricted therapeutic option. However, its potential benefit should be interpreted with caution in light of the overall poor prognosis and small sample size. Earlier diagnosis, management of comorbidities, and prompt intervention may help improve outcomes in selected patients.

## Supplementary Information

Below is the link to the electronic supplementary material.

Supplementary Material 1

## Data Availability

The datasets generated and/or analyzed during the current study are available from the corresponding author on reasonable request.
