# Supplementary material for: Allogeneic Hematopoietic Cell Transplantation for Congenital Athymia: A Nationwide Retrospective Study in Japan
Source: J Clin Immunol. 2026 Mar 28;46(1):47. doi: 10.1007/s10875-026-02008-y (PMC13149597; doi:10.1007/s10875-026-02008-y)
Supplement: Supplementary file 1 — Supplementary Material 1 [file 10875_2026_2008_MOESM1_ESM.docx]

**Supporting Information**

*Journal of Clinical Immunology*

**Allogeneic Hematopoietic Cell Transplantation for Congenital Athymia: A Nationwide Retrospective Study in Japan**

Tsubasa Nishinosono, MD^1,2^, Hideki Muramatsu, MD, PhD^†1^, Manabu Wakamatsu, MD, PhD^1^, Motoshi Sonoda, MD, PhD^3^, Katsuhide Eguchi, MD, PhD^3^, Koji Kawaguchi, MD, PhD^4^, Takeshi Yamamoto, MD, PhD^5^, Takahiro Kudo, MD, PhD^6^, Michiko Kajiwara, MD, PhD^7^, Masataka Ishimura, MD, PhD^3^, Yoshiyuki Takahashi, MD, PhD^†1^

^1^Department of Pediatrics, Nagoya University Graduate School of Medicine, Nagoya, Japan

^2^Department of Pediatric Critical Care, Shizuoka Children's Hospital, Shizuoka, Japan

^3^Department of Pediatrics, Graduate School of Medical Sciences, Kyushu University, Fukuoka, Japan

^4^Department of Hematology and Oncology, Shizuoka Children’s Hospital, Shizuoka, Japan

^5^Department of Pediatrics, Graduate School of Medicine, Chiba University, Chiba, Japan

^6^Department of Pediatrics, Juntendo University Faculty of Medicine, Tokyo, Japan

^7^Center for Transfusion Medicine and Cell Therapy, Institute of Science Tokyo Hospital, Tokyo, Japan

**Supplemental Appendix pp. 2**

**Supplemental Appendix.** Lists of Surveyed Institutions

**Supplemental Tables pp. 3–5**

**Table S1.** Congenital comorbidities

**Table S2.** Laboratory characteristics

**Table S3.** Post-transplant immunoglobulin replacement therapy (IRT) and vaccine-induced antibody response

**Supplemental Appendix. Lists of Surveyed Institutions**

All of the following centers provide care for inborn errors of immunity or autoinflammatory disease patients and collaborated in this study: Aichi Medical University Hospital, Akita University Hospital, Asahikawa Medical University Hospital, Chiba Children's Hospital, Chiba University Hospital, Dokkyo Medical University Hospital, Dokkyo Medical University Saitama Medical Center, Ehime University Hospital, Fujita Health University Hospital, Gifu University Hospital, Gunma University Hospital, Hirosaki University Hospital, Hiroshima University Hospital, Hokkaido University Hospital, Hospital of the University of Occupational and Environmental Health, Hyogo Prefectural Kobe Children's Hospital, Hyogo Medical University Hospital, Institute of Science Tokyo Hospital, Iwate Medical University Hospital, Japanese Red Cross Aichi Medical Center Nagoya Daiichi Hospital, Jichi Medical University Hospital, Juntendo University Hospital, Kagawa University Hospital, Kagoshima University Hospital, Kanagawa Children's Medical Center, Kanazawa University Hospital, Kansai Medical University Hospital, Kitasato University Hospital, Kobe University Hospital, Kochi University Hospital, Kurume University Hospital, Kyoto Prefectural University of Medicine Hospital, Kyoto University Hospital, Kyushu University Hospital, Mie University Hospital, Miyazaki University Hospital, Nagano Children's Hospital, Nagasaki University Hospital, Nagoya University Hospital, Nara Medical University Hospital, National Center for Child Health and Development, National Center for Global Health and Medicine Hospital, National Defense Medical College Hospital, Niigata University Medical & Dental Hospital, Oita University Hospital, Okayama University Hospital, Osaka City General Hospital, Osaka Women’s and Children’s Hospital, Osaka Medical and Pharmaceutical University Hospital, Osaka Metropolitan University Hospital, Osaka University Hospital, Saga University Hospital, Shiga University of Medical Science Hospital, Shimane University Hospital, Shinshu University Hospital, Shizuoka Children's Hospital, St. Luke's International Hospital, Tohoku University Hospital, Tokyo Medical University Hospital, Tokyo Metropolitan Children's Medical Center, Tottori University Hospital, University of Fukui Hospital, University of Tsukuba Hospital, University of Yamanashi Hospital, Wakayama Medical University Hospital, Wakayama Red Cross Hospital, Yamagata University Hospital, Yokohama City University Hospital.

**Table S1. Congenital comorbidities**

| UPN | Congenital heart diseases | Airway disorders | HypoPTH | Dysmorphic  face | Coloboma | Neuropsychiatric disorders | Other anomalies |
| --- | --- | --- | --- | --- | --- | --- | --- |
| 1 | – | – | Y | Y | – | Enceph. | – |
| 2 | TA, IAA, ARSA | TBM, CA | Y | Y | Y | Ep, Deaf., ID | Polydactyly |
| 3 | PDA | TBM | Y | Y | Y | Ep, Deaf., ID | – |
| 4 | ARSA, RAA | MG | Y | Y | – | – | – |
| 5 | ASD | TBM, LM | Y | Y | Y | CD, FP, ID | Dysphagia, esophageal atresia, gastroesophageal reflux disease, oligodactyly, and short stature |
| 6 | VSD | NPA | Y | Y | – | Ep, ID | Thrombocytopenia and short stature |
| 7 | HLHS, CoA, VSD, ASD | TBM, CP | Y | Y | Y | Ep, Deaf., ID | Dysphagia |
| 8 | PDA | TBM, CP | Y | Y | Y | CBD, Deaf., ID | Dysphagia |
| 9 | PDA | MG, GP, NS | Y | Y | Y | Ep, CD, Deaf., ID | Esophageal hiatus hernia, polydactyly, and short stature |

ARSA, aberrant right subclavian artery; ASD, atrial septal defect; CA, choanal atresia; CBD, cerebellar dysplasia; CD, cerebral dysplasia; CoA, coarctation of the aorta; CP, cleft palate; Deaf., deafness; Enceph., encephalopathy; Ep, epilepsy; FP, facial palsy; GP, glossoptosis; HLHS, hypoplastic left heart syndrome; HypoPTH, hypoparathyroidism; IAA, interrupted aortic arch; ID, intellectual disability; LM, laryngomalacia; MG, micrognathia; NPA, nasopharyngeal atresia; NS, nasal stenosis; PDA, patent ductus arteriosus; RAA, right aortic arch; TA, truncus arteriosus; TBM, tracheobronchomalacia; UPN, unique patient number; VSD, ventricular septal defect.

**Table S2. Laboratory characteristics**

| Characteristics | Patients (*n* = 9) |
| --- | --- |
| **Pre-transplant immunological data, median (range)** |  |
| Lymphocytes, ×10^9^/L | 1.253 (0.303–3.120) |
| CD3^+^ T-cells, ×10^9^/L | 0.026 (0–0.080) |
| CD4^+^ T-cells, ×10^9^/L | 0.003 (0–0.071) |
| CD8^+^ T-cells, ×10^9^/L | 0.001 (0–0.065) |
| CD19^+^ B-cells, ×10^9^/L | 0.515 (0.158–2.028) |
| CD56^+^ NK T-cells, ×10^9^/L | 0.262 (0.021–0.510) |
| CD45RA^+^ naïve T-cell, ×10^9^/L | 0 (0–0) |
| CD45RO^+^ memory helper T-cell, ×10^9^/L | 0 (0–0.070) |
| IgG, g/L | 3.15 (0.74–12.05) |
| IgA, g/L | 0.06 (0.01–0.57) |
| IgM, g/L | 0.29 (0.19–0.84) |

**Table S3. Post-transplant immunoglobulin replacement therapy (IRT) and vaccine-induced antibody responses**

| UPN | Post-transplant IRT | IRT duration post-HCT, months | Antibody response to vaccination |
| --- | --- | --- | --- |
| 2 | Y | 9 | - |
| 3 | Y | NA | - |
| 6 | - | - | Diphtheria, polio, pertussis |
| 7 | Y | 11 | HBsAg |
| 8 | Y | 36 | - |
| 9 | - | - | HBsAg |

HBsAg, hepatitis B surface antigen; HCT, hematopoietic cell transplantation; IRT, immunoglobulin replacement therapy; NA, not available; UPN, unique patient number
